# 

**Published:** 1858-11

**Authors:** 


### CONTENTS.

***ORIGINAL COMMUNICATIONS.***

PAGE.

ARTICLE I.—Use of Opium in Children. By THOMAS POLLARD, M. D., Richmond, Va., ........ 129

ARTICLE II.—Sulphate of Zinc in Hemorrhages. By WM. HAUSER, M. D., of Speir's Turnout, Ga., ........ 135

***SELECTIONS.***

Researches into the Types of Diseases and Types of Therapy. By Bennet Dowler, M. D., ........ 137

An Epistle upon Yellow Fever, ........ 157

Progress of Medical Science—Selections from Foreign Journals—On the treatment of Croup, ........ 163

On Local Anæsthesia and Electricity, ........ 167

On Inflamed Nipples during Lactation, ........ 172

The Uterus, ........ 173

Rational Medicine—A Review, ........ 178

***EDITORIAL AND MISCELLANEOUS.***

Medical Ethics, ........ 184

Nashville Monthly Record of Medical and Physical Science, ........ 185

The Medical and Surgical Reporter, Philadelphia, Penn., ........ 185

Publications Received, ........ 186

Professional Etiquette, ........ 187

News Items—Medical Facts, &c., ........ 189

The Georgia Temperance Crusader for 1859, ........ 192

